# A Comparative Study of the Graston Technique and Alfredson Protocol in the Management of Achilles Tendinopathy

**DOI:** 10.7759/cureus.62249

**Published:** 2024-06-12

**Authors:** Aliyah Usman Qureshi, Muddsar Hameed, Muhammad Umar, Umer Yasir, Zamurd Abbas, Sarem Jamil, Linta Masroor, Arusa Arshad, Saima Tahir, Muhammad Ibrahim Raza

**Affiliations:** 1 Department of Physiotherapy, Rawalpindi Medical University, Rawalpindi, PAK; 2 Department of Clinical Psychology, Shifa Tameer-e-Millat University, Islamabad, PAK; 3 Department of Medicine, Shifa Tameer-e-Millat University, Islamabad, PAK; 4 Department of Medicine, International European University, Bishkek, KGZ; 5 Department of Anatomy, Khyber Medical College, Peshawar, PAK; 6 Department of Orthopaedics, Shifa Tameer-e-Millat University, Islamabad, PAK; 7 Department of Medicine, Fazaia Medical College, Islamabad, PAK; 8 Department of Medicine, Royal Metropolitan University, Bishkek, KGZ

**Keywords:** pain, calf muscle strength, alfredson technique, graston technique, achilles tendinopathy

## Abstract

Background and objectives: The Achilles tendon, the largest and strongest tendon in the human body, is frequently injured by overuse; this condition is known as Achilles tendinopathy (AT). It serves as a link between the heel bone and the calf muscles and is necessary for motions, such as walking, sprinting, and jumping. Evidence is presented to support the efficacy of the Graston technique and Alfredson protocol for pain reduction and improvement of function and calf muscle strength. The objective of this study is to compare the efficacy of the Graston technique versus the Alfredson protocol in patients with AT.

Methods and data collection: After obtaining approval from the ethical review board of the Rawalpindi Medical University, all patients fulfilling the inclusion criteria are divided into two groups, A and B, by generating random identity numbers using Microsoft Excel for allocation. Group A comprises patients who undergo treatment with the Graston technique as conventional therapy with Alfredson protocol (12-week calf muscle eccentric exercises), while those in group B follow a Graston technique with sole heel lift. Individuals in the eccentric exercises group follow an Alfredson method-based 12-week eccentric exercise plan for their leg muscles. The workouts need to be done twice a day, seven days a week for 12 weeks. The plan includes two exercises: the first done with the knee straightened to work the gastrocnemius and the second done with the knee bent to work the soleus. Three sets of 15 repetitions with no rest interval for each exercise are completed twice a day on the affected limb to yield functional improvement.

Results: The results showed that both the Alfredson protocol and the Graston technique were effective in managing AT symptoms. The study involved dividing 32 participants into two groups who received either treatment for four weeks. The main way to measure improvement was a score called the Villalta-Scanlon Achilles Tendonitis Index score. In both groups, these scores showed significant improvement (with a p-value less than 0.001, which means that the results are very statistically significant). For Group A (who received the Alfredson protocol), the average Villalta-Scanlon Achilles Tendonitis Index score before treatment was 29.25. This score increased to 31.25 at mid-treatment and 34.38 after the full four weeks of treatment. Group B (who received the Graston technique) started with an average Villalta-Scanlon Achilles Tendonitis Index score of 22.94. Their scores also increased throughout the treatment, reaching 34.94 at mid-treatment and 42.88 after four weeks. These findings provide evidence that both treatments can improve AT symptoms, with some suggestions that the Graston technique might be even more effective based on the higher average Villalta-Scanlon Achilles Tendonitis Index scores after treatment.

Conclusions: The Graston technique shows promising results, particularly in the mid- and post-treatment phases, indicating its potential efficacy in comparison to the Alfredson protocol in the treatment of AT.

## Introduction

The human Achilles tendon is the strongest in the body. The calcaneus is connected to the gastrocnemius and soleus calf muscles by a strong ring of fibrous tissue. By pulling on the heel, the Achilles tendon enables us to stand on our toes. Tendinitis is the term for Achilles tendon inflammation. As a result, any type of tendon pathology is now referred to as tendinopathy rather than tendinitis. Given the literature analysis, it appears that this disorder lacks inflammatory infiltrates, suggesting that anti-inflammatory drugs may not be necessary for its treatment. However, to alleviate pain and swelling, treatment typically involves rest, ice, elevation, and possibly anti-inflammatory drugs. Physical therapists (PTs) frequently suggest exercises to enhance flexibility and strengthen the tendon. In extreme circumstances, tendon restoration may require surgery [1].

Two Achilles tendinopathies (ATs) are particularly prevalent in individuals aged 30-60. The Graston technique is a manual therapy (MT) method that mobilizes soft tissues with the use of stainless-steel tools. It is frequently used to treat tendinopathies, such as ATs and other musculoskeletal problems. Nevertheless, there is insufficient evidence to support the effectiveness of the Graston technique in treating ATs. According to certain research, people with tendinopathies, especially ATs, may find relief from pain and improved function using manual therapies like the Graston technique. To compare its efficacy to alternative treatments such as the Alfredson technique, more research is necessary [2].

Depending on the population being researched and the description of the ailment, the prevalence of ATs varies. The overall incidence of AT is estimated to be 1.85 cases per 1,000 patients, with a higher rate of 2.35 cases per 1,000 in the 21-60-year-old population [3]. The estimated incidence in the general population is 2-6%, with greater percentages noted in particular groups including athletes, runners, and members of the armed forces. AT is more common in runners; it can affect elite runners up to 52% of the time, compared to recreational runners’ 5-12% incidence. Up to 9% of athletes play jumping and running sports, such as basketball and volleyball, where the incidence is also higher [4].

An analysis conducted in 2017 estimated that 20% of instances of ATs were caused by insertional tendinopathy. According to a different 2019 study, 25% of the cases in the study population had insertional AT (AIT). Conversely, non-insertional AT (NIAT) is thought to occur more frequently. In a 2020 study, Maffulli, Longo, and Kadakia found that in the population they studied, NIAT accounted for almost 77% of all occurrences of the condition. In general, AIT is thought to be less prevalent than NIAT; nevertheless, the precise prevalence of each kind can differ based on the population under investigation and the diagnostic standards applied [5].

The anatomy of the Achilles tendon consists of multiple essential elements. Collagen fibers, which offer strength and flexibility, make up the majority of its composition. A thin sheath known as the paratenon envelops the tendon and aids in reducing friction during movement. There is not much blood flow to the Achilles tendon, particularly in the center, which may make it more difficult for the tendon to repair effectively after an injury, leading to the development of tendinopathy. At the calcaneal tuberosity, the Achilles tendon inserts into the calcaneus, the rear of the heel bone. This region may be impacted by AIT and is susceptible to degenerative alterations [6].

AT is caused by overuse or overload stresses, which lead to repetitive microtrauma. AT is becoming more common and yet confuses medical professionals worldwide. There are two types of AT, insertional and NIAT. According to recent research, tendinopathy may result from extrinsic risk factors, such as medication use that interacts with tendon metabolism and mistakes made during training and work-related tasks. The risk of AT is raised when combined with intrinsic risk factors, anomalies in the biomechanics of the lower leg, and metabolic and inflammatory diseases, such as hypercholesterolemia, diabetes, and genetic aberrations [7].

Patients may describe a history of Achilles tendon overuse or repetitive strain from physical activity, such as running, leaping, or other exercises. They might also bring up a recent increase in activity or footwear modification. Patients who have AT usually complain of pain near the tendon’s midpoint or where it inserts into the calcaneus. Patients typically report functional impairment during load-bearing activities, focal discomfort, and localized tendon thickening. Patients frequently feel stiffness or soreness in the morning when they wake up or at the start of an activity session. The pain might go away if the patient keeps moving, but in long-term situations, discomfort may be constant and occur even while at rest. Depending on the degree and duration of the ailment, patients with AT may present differently clinically. However, they often exhibit a few standard signs and symptoms [8]. The primary sign of AT is pain, which is typically experienced in the Achilles tendon itself, especially in the insertional tendinopathy area where the tendon inserts into the heel bone or in the non-insertional tendinopathy area where the tendon is midway. A dull aching or stiffness that gets worse with movement and sometimes gets better with rest is how the discomfort is commonly characterized. Particularly in cases of acute or severe tendinopathy, there may be Achilles tendon swelling. Usually, the swelling only affects the portion of the tendon that is afflicted [9].

AIT is diagnosed by tendon probing in cases of discomfort. Diagnosis of tendinopathy can be made in patients with AT if they have a tender area of intra-tendinous swelling that moves with the tendon and whose tenderness significantly reduces or disappears when the tendon is put under tension. There is also a high positive predictive chance that the tendon will exhibit histologic and ultrasonographic features of tendinopathy. Symptoms experienced by the patient, such as the source and length of the pain, any aggravating or mitigating circumstances, and any pertinent past injuries or illnesses, point to the clinical presentation of a patient suffering from AT. Physical inspection of the Achilles tendon and feeling for evidence of thickness, warmth, soreness, or swelling, help in reaching a diagnosis. Measurement of the ankle joint’s range of motion and testing the calf muscles’ strength also help clinicians to diagnose. To determine the degree of tendon injury and to confirm the diagnosis of AT, imaging tests such as MRIs and ultrasounds may be requested.

Pharmacological therapy is effective in the symptomatic management of AT. Nonsteroidal anti-inflammatory drugs may prove effective in relieving the symptoms or reducing the pain. These medications act as the first line of therapy, being easily available and affordable and having acute effects. The use of oral steroids and treatment with steroid injections are often used to manage acute episodes but may be followed by complications and side effects due to long-term use. Several conservative treatment modalities have been proposed in the past few years for AT, with local drug administration, such as blood products, hyaluronic acid, botulinum toxin, polidocanol, proteinase inhibitors, corticosteroids, and high-volume image-guided injections playing an increasingly important role [10].

The use of orthotics to obtain immobilization, resulting in minimizing motion, may prove beneficial in relieving symptoms. The electrotherapeutic modalities used in the treatment of AT include ultrasound and infrared treatment and the use of electrical stimulations including transcutaneous electrical nerve stimulation (TENS) and interferential therapy (IFT). These can be combined with heat or ice for better effects. MT can play a key role in treating the concerned condition [11]. To help relax muscular tension, enhance blood flow, and encourage Achilles tendon repair, manual therapies such as massage, myofascial release, and instrument-assisted soft tissue mobilization are used in this procedure. By using mild, manual approaches to increase the ankle joints’ and surrounding joints’ mobility, patients can lessen the strain on the Achilles tendon and enhance general function. Particular stretches for the Achilles tendon and the gastrocnemius and soleus muscles of the calf can help release tension from the tendon and increase flexibility. The Achilles tendon can be made stronger and more resilient by performing progressive strengthening activities for the calf muscles and the muscles of the foot and ankle.

The tensioning technique of neural mobilizations involves the displacement of nerve endings in opposite directions, which results in the elongation of the nerve bed. The tensioner is a neurodynamic test that produces an increase in tension in neural structures. It relies on the natural viscosity of the neural system and does not cross the elastic limit. Therefore, this technique is not harmful to the nervous bed, and when applied gently, it may improve neural viscoelastic and physiological functions [12].

In 2017, Habets et al. conducted a randomized controlled trial to evaluate the efficacy of Alfredson versus Silbernagel exercise therapy in treating midportion AT. Eighty-six recreational athletes (21-60 years of age) with unilateral chronic midportion AT (i.e., ≥3 months) were included in this multicenter assessor-blinded randomized controlled trial. They were randomly allocated to either a group performing the Alfredson isolated eccentric training program (n = 43) or a group performing the Silbernagel combined concentric-eccentric program (n = 43). The results of this study enlarged the evidence base on different exercise programs for AT and may aid the clinician in choosing the most appropriate program for their patients. Clinicians prefer Alfredson over Silbernagel as a better treatment choice [13].

In 2019, Kawin et al. performed a controlled trial to compare the Achilles tendon load during running in flatfeet participants using customized arch support orthoses versus an orthotic heel lift (HL). Twelve participants were recruited and ran along the runway in the laboratory for three conditions: (1) without orthoses, (2) with customized arch support orthoses (CASO), (3) with orthotic HL. The researchers deduced that both CASO and HL were able to cause a significant reduction in peak Achilles tendon load (ATL) and Achilles tendon load rate (ATLR) compared to those without orthotic conditions [14].

In 2021, Chantel et al. conducted a study on the efficacy of HLs versus calf muscle eccentric exercise (ECC) on mid-portion AT. One hundred participants (52 women and 48 men, mean age 45.9, SD 9.4 years) with clinically diagnosed and ultrasonographically confirmed mid-portion AT were randomly allocated to either an (1) HL (n = 50) or (2) ECC (n = 50) group. The researchers concluded that in adults with mid-portion AT, HLs were more effective than calf muscle ECC in reducing pain and improving function at 12 weeks [15].

Rationale

The rationale for this study is to address the existing gap in the literature by conducting a comparative analysis of the Graston technique and Alfredson protocol. Given the increasing adoption of the Graston technique in clinical settings, it is crucial to evaluate its effectiveness in managing AT rigorously. This study aims to provide evidence-based insights into the relative benefits of these two treatment modalities, thereby guiding clinicians in selecting the most appropriate intervention for patients with AT. Moreover, this study holds significant relevance for the Pakistani context, where the burden of musculoskeletal disorders is substantial, and access to diverse therapeutic options may be limited. By investigating the efficacy of these treatments in a local healthcare setting, the study seeks to enhance clinical practice and improve patient outcomes in managing AT. The findings will contribute to the broader body of knowledge on AT treatment, potentially influencing guidelines and recommendations for managing this condition.

## Materials and methods

We employed a comparative study design to investigate the efficacy of the Graston technique versus the Alfredson protocol in patients with AT. The research was conducted at the physiotherapy outpatient departments of various hospitals including Holy Family Hospital, Benazir Bhutto Hospital, and District Headquarter Hospital in Rawalpindi, India. The study spanned over six months after the approval from IRB Rawalpindi Medical University (IRB #057-23). A sample size of 32 patients, with 16 individuals allocated to each treatment group, was determined using the OpenEpi sample size calculator (OpenEpi: Open Source Epidemiologic Statistics for Public Health, www.OpenEpi.com). Nonprobability convenient sampling was utilized for patient enrollment based on predefined inclusion criteria, which included age between 18 and 65 years, diagnosis of AT, decreased plantar flexor strength, insidious onset of pain aggravated by weight-bearing activity, symptoms lasting more than three months, and others. Exclusion criteria were also established to ensure the homogeneity of the sample population. Data collection tools comprised the Visual Analogue Scale (VAS) for pain assessment, the Victorian Institute of Sports Assessment (VISA-A) for functional evaluation, and manual muscle testing. These meticulously chosen methodologies and criteria facilitated a comprehensive investigation into the comparative efficacy of the two treatment protocols, providing valuable insights into the management of AT.

Data collection

After we obtained approval from the ethical review board of the university, all patients fulfilling the inclusion criteria were divided into two groups, A and B, by generating random identity numbers using Microsoft Excel (Microsoft Corporation, USA) for allocation. Group A comprised patients who underwent treatment with the Alfredson protocol (12-week calf muscle ECC), while those in group B followed the Graston technique. Individuals in the ECC group followed an Alfredson method-based 12-week ECC plan for their leg muscles. The workouts needed to be done twice a day, seven days a week for 12 weeks. People who had signs on both sides did the course on both sides. The plan included two exercises: the first was done with the knee straightened to work the gastrocnemius, and the second was done with the knee bent to work the soleus. Three sets of 15 repetitions with no rest interval between each exercise were done twice a day on the affected limb(s). Initially, gravity was used as support, and people would stand with all their weight on the wounded leg. The participants were told to continue with the workout even if it hurt. When people could do both movements on their hurt side without pain or soreness, they were told to increase the resistance part of the plan to strengthen the calf muscles. The participants were instructed to use a weighted bag to improve resistance. They were to start by adding 5 kg of weight to the pack and then add 5 kg more each time as needed. Group B underwent soft tissue mobilization with Graston on the affected Achilles tendon. This category of patients attended the clinic twice per week for 12 weeks. During each session, a progression of instruments with decreasing areas of contact was used. Each treatment session lasted approximately 10-15 minutes and was used to evaluate the decrease in pain and functional improvement. Data regarding age, gender of the participants, side involved, and Victorian Institute of Sport Assessment-Achilles (VISA-A) questionnaire score were recorded on a proforma. The patients were followed up at zero, two, and four weeks of treatment. To minimize bias, the study incorporated single-blinding, where the participants were unaware of their group allocation. Blinding was achieved by using a separate team member, who was not involved in the treatment administration, to conduct the randomization and allocation process. In addition, the data collection and assessment were performed by blinded assessors who were unaware of the participants' group assignments, ensuring unbiased outcome evaluation.

Data analysis

Data were entered and analyzed using IBM SPSS Statistics for Windows, version 23.0 (released 2015, IBM Corp., Armonk, NY). Quantitative variables such as the age of the participants and VISA-A questionnaire score were represented in mean ± S.D. Qualitative variables such as gender and side involved were represented in frequencies and percentages. A paired t-test was applied to compare qualitative variables between the groups. A p-value of ≤0.05 was considered a significant association.

## Results

The following section provides a detailed presentation and interpretation of the results obtained from this comparative study, shedding light on the comparative efficacy of the Graston technique with the Alfredson protocol versus the Graston technique with a sole HL in patients with AT.

Table 1 shows a sociodemographic profile of the study’s 32 participants, revealing a diverse distribution across various variables. Age-wise, 40% of the individuals were between 18 and 35 years, whereas 43% fell within the 36-50 age groups, and 15% were aged 51-65. Gender distribution showed that 69% of the participants were male and 31% were female. Body mass index (BMI) classifications indicated that 34% of the individuals were within the healthy weight range, 56% were overweight, and 9% were classified as obese. Regarding marital status, 75% of the participants were married, whereas 25% were single. These descriptive statistics offer a comprehensive snapshot of the study’s participant demographics, encompassing age, gender, BMI, and marital status within this sample population.

**Table 1:** Descriptive statistics of sociodemographic variables (N = 32) *f* stands for the number of patients.

Variables	f	%
Age		
18–35	13	40
36–50	14	43
51–65	5	15
Gender		
Male	22	69
Female	10	31
Marital status		
Married	24	75
Single	8	25
BMI		
Health weight range	11	34
Overweight range	18	56
Obese range	3	9

Table 2 shows the mean comparison of the effectiveness of the Alfredson protocol versus the Graston technique conducted using the VISA-A and VAS as assessment tools. The data, organized into pretreatment (one week), mid-treatment (two weeks), and posttreatment (four weeks) phases for both groups (A and B), provide valuable insights into the trends of these interventions. In the pretreatment phase, Group A (Alfredson protocol) exhibited a mean VISA-A score of 29.25, whereas Group B (Graston technique) had a slightly lower mean of 22.94. As the treatment progressed, the mid-treatment phase showed an increase in mean VISA-A scores for both groups, with Group A reaching 31.25 and Group B surpassing a mean of 34.94. The posttreatment phase further demonstrated improvement, where Group A’s mean VISA-A score elevated to 34.38, whereas Group B exhibited a higher mean of 42.88. Regarding pain assessment using the VAS, Group A started with a mean of 6.94 in the pretreatment phase, which remained consistent in the mid-treatment phase. By contrast, Group B showed a slight reduction from 6.06 to 5.06 during the same period. In the posttreatment phase, both groups demonstrated a decrease in pain levels, with Group A reaching a mean VAS score of 5.94 and Group B further reducing to 4.06. These findings suggest that both interventions contributed to the improvement of AT symptoms throughout the study. The Graston technique (Group B) shows promising results, particularly in the mid- and posttreatment phases, indicating its potential efficacy in comparison to the Alfredson protocol (Group A).

**Table 2:** Mean comparison of the effectiveness of the Alfredson protocol versus the Graston technique Group A: Patients treated with Alfredson technique. Group B: Patients treated with Graston technique. LL: lower limit, UL: upper limit

	Group	Mean	Std. deviation	Std. error mean	P	LL	UL	Cohen's d
VISA_A_Pre	Group A	29.25	12.157	3.039	0.000	14.8 -	24.3 -	1.48
	Group B	22.94	12.476	3.119	0.001			
VISA_A_Mid	Group A	31.25	11.958	2.990	0.000	21.1	32.2	1.91
	Group B	34.94	14.535	3.634	0.000			
VISA_A_Post	Group A	34.38	10.145	2.536	0.001	28 .1	38.1	2.56
	Group B	42.88	12.764	3.191	0.000			
VAS_Pre	Group A	6.94	1.389	0.347	0.000	14.8	24.3	1.48
	Group B	6.06	1.569	0.392	0.000			
VAS_Mid	Group A	6.94	1.389	0.347	0.000	21.1	32.1	1.91
	Group B	5.06	1.569	0.392	0.000			
VAS_Post	Group A	5.94	1.389	0.347	0.000	28.1	38.1	2.56
	Group B	4.06	1.569	0.392	0.000			

Tables 3-4 show that paired sample t-tests were conducted to assess the effectiveness of the two treatment protocols (Alfredson protocol for Group 1 and Graston technique for Group 2) on the participants with AT. The VISA-A scale was utilized for the assessment, and the results indicate significant changes in the mean scores throughout the treatment. For Group 1 (Alfredson protocol), the paired sample t-test revealed a statistically significant decrease in VISA-A scores from the pretreatment phase (mean = 29.25) to the mid-treatment phase (mean = 31.25) and further to the posttreatment phase (mean = 34.38). The associated p-values for all pairs were highly significant (p < 0.001), suggesting a substantial improvement in the condition. Similarly, for Group 2 (Graston technique), the paired sample t-test demonstrated a significant increase in VISA-A scores from the pretreatment phase (mean = 22.94) to the mid-treatment phase (mean = 34.94) and further to the posttreatment phase (mean = 42.88). The p-values for all pairs were highly significant (p < .001), indicating a notable improvement in AT symptoms with the Graston technique. In summary, both treatment groups exhibited significant changes in VISA-A scores over the treatment period, suggesting improvements in the condition of AT. These findings provide evidence for the efficacy of both the Alfredson protocol and Graston technique in managing and alleviating symptoms associated with this condition. 

**Table 3:** Paired sample t-test results of the Alfredson protocol

		Mean	N	Std. deviation	Std. error mean	P
Pair 1	Group	1.00	16	.000	0.000	0.01
	Pre (VISA-A)	29.25	16	12.157	3.039	0.00
Pair 2	Group	1.00	16	0.000	0.000	0.00
	Mid (VISA-A)	31.25	16	11.958	2.990	0.01
Pair 3	Group	1.00	16	0.000	0.000	0.00
	Post (VISA-A)	34.38	16	10.145	2.536	0.00

**Table 4:** Paired sample t-test results of the Graston technique

		Mean	N	Std. deviation	Std. error mean	p
Pair 1	Group	2.00	16	0.000	0.000	0.01
	Pre (VISA-A)	22.94	16	12.476	3.119	0.00
Pair 2	Group	2.00	16	0.000	0.000	0.00
	Mid (VISA-A)	34.94	16	14.535	3.634	0.01
Pair 3	Group	2.00	16	0.000	0.000	0.001
	Post (VISA-A)	42.88	16	12.764	3.191	0.00

The results demonstrate that both treatment groups exhibited significant changes in VISA-A scores over the treatment period, suggesting improvements in the condition of AT. These findings provide evidence that the efficacy of the Graston technique is more significant as compared to the Alfredson protocol in managing and alleviating symptoms associated with AT.

## Discussion

The study was conducted to compare the effectiveness of the Graston technique versus the Alfredson protocol on pain and functional abilities in patients suffering from AT. Although a large body of research has demonstrated the benefits of both soft tissue mobilization and ECC on individual variables, there are relatively few studies whose authors compare the two approaches in tandem. In addition, the effectiveness of both methods in treating AT was the topic of the current study. The readings were taken as pretreatment readings at the beginning of the treatment protocol and again as posttreatment readings at the end of the second or fourth week [16].

A prospective comparative study evaluating the effectiveness of eccentric training (ECC) versus both ECC and soft tissue treatment in the treatment of Achilles insertional tendinopathy (AIT) was presented by McCormack et al. in 2016. A total of 16 participants were randomized to receive either ECC alone or combination of soft tissue therapy (Astym). The intervention was carried out over 12 weeks, and at baseline, four, eight, 12, 26, and 52 weeks, the outcomes were evaluated. The global rating of change, the numerical pain rating scale, and the VISA-A were among the outcomes. The researchers concluded that, for both short- and long-term follow-up periods, ECC alone was less successful than soft tissue therapy (Astym) + ECC at improving function. Similarly, the present research indicates that soft tissue mobilization has a better outcome as compared to ECC [17].

In 2016, Jayaseelan studied MT and ECC in the management of AT. Although the care of AT has not been researched, MT has shown promising results in the treatment of other chronic tendon dysfunctions, such as lateral epicondylalgia. For chronic AT, three runners saw a PT. Calf stretches and eccentric loading exercises were advised. The inclusion of MT targeted at both local and distant locations in this study yielded AT patients’ recovery. However, the limitation was that incorporating joint mobilization into routine AT therapy requires more investigation [18].

Christenson conducted research in 2007 on the efficacy of particular soft tissue mobilizations in the treatment of Achilles tendinitis, and it was an experimental design single-case study. The subject of the investigation was a 39-year-old woman who had Achilles tendinitis for five years. The study comprised three six-week periods: the pretreatment, treatment, and posttreatment phases. Although the single-case research design restricts extrapolation, the outcomes are consistent with the use of specific soft tissue mobilization (SSTM) to treat Achilles tendinosis. This supports my study in the way it shows the effectiveness of soft tissue mobilization in treating AT [19].

Pressure massage was used in 2019 by Stefansson et al. to treat AT. This was a randomized controlled single-blind trial in which the researcher contrasted an ECC protocol with a novel treatment. Three groups of 60 AT patients were randomly assigned to different treatments: Group 1 undertook an ECC routine, Group 2 received pressure massage, and Group 3 received both the ECC protocol and pressure massage. One effective treatment for AT is pressure massage when compared to eccentric activity therapy, pressure massage produces comparable outcomes. The author concluded that the results were not improved by combining the two therapies. This provides some support for my research by showing the efficacy of ECC for AT patients [20].

A study on the treatment of AT using heavy slow resistance (HSR) versus eccentric training was conducted in 2015 by Beyer et al. in the form of a randomized controlled trial. Twelve weeks of eccentric training (ECC) treatment was randomized for 58 individuals with midportion AT that was persistent (>3 months). The study’s findings demonstrated that patients with AT could benefit from both HSR and traditional ECC and that the latter is generally linked to better patient satisfaction at 12 weeks but not at 52 weeks. This showed that ECC was a useful treatment for AT, but the authors omitted information about the impact of soft tissue mobilization [21].

Zhi et al. (2021) investigated the nonoperative management of AIT. In this study, 23 papers with 35 groups were considered. The authors concluded that extracorporeal shockwave therapy or the combination of extracorporeal shockwave therapy and ECC is the preferred nonoperative treatment option for AIT based on available data. Similarly, they found the same supporting evidence of ECC in the treatment of AT [22]. Kim et al. conducted a study in 2017 on the therapeutic efficacy of instrument-assisted soft tissue mobilization for soft tissue injury. They found that similar to this research, soft tissue mobilization has highly promising results in the treatment of AT, a soft tissue injury [23].

The sociodemographic profile of the 32 study participants offers important information about the makeup of the sample population. The age distribution shows a rather equal presence across age categories, with the 36-50 age group having a slight majority. This distribution raises the possibility that a wide age range of adults may benefit from the study’s conclusions. Many studies have shown a consistent pattern in the gender distribution, with a higher percentage of male participants. This trend may be due to underlying gender differences in the prevalence of specific illnesses or in the habit of seeking health care. Notable is the distribution of BMI categories, showing that most participants are overweight. This discovery could have an impact on the study, especially if BMI is a factor under investigation or if it affects the outcomes under assessment [24].

Given that marital status might occasionally affect health behaviors and outcomes, the study’s findings may potentially be affected by the distribution of participants’ marital status, the majority of whom were married. Overall, the study’s conclusions may apply to a wide spectrum of adults based on the varied distribution observed across various sociodemographic characteristics. When analyzing the study’s wider implications and interpreting the findings, it is crucial to take these demographic characteristics into account. The VISA-A and VAS scores are used to compare the efficacy of the Alfredson regimen and Graston technique in treating AT. The results show some intriguing patterns in how each approach affects symptoms over time. Group B (Graston technique) and Group A (Alfredson procedure) began the pretreatment phase with similar VISA-A scores, although Group B’s mean was somewhat lower. This implies that baseline levels of AT symptoms were being addressed at similar degrees by both therapies.

The VISA-A scores of both groups improved as the treatment went on. Nonetheless, Group B continuously performed better than Group A, as evidenced by higher mean VISA-A scores during the mid- and posttreatment stages. This suggests that the Alfredson technique may not be as successful as the Graston technique in improving functional results associated with AT [25].

Using the VAS to measure pain, both groups’ pain levels decreased over the duration of the treatment. In comparison to Group A, Group B showed a more notable decrease in pain, especially during the posttreatment phase. This implies that, in comparison to the Alfredson protocol, the Graston technique may also be more successful in easing AT-related discomfort. These results imply that the Graston technique and Alfredson procedure are both useful treatments for reducing AT symptoms. However, the Graston technique seems to be more effective, especially when it comes to increasing functional results and lowering discomfort levels. Larger sample sizes and longer follow-up times may be necessary for future studies to validate these results and investigate the mechanisms behind the observed variations in the two therapies’ efficacies [26].

The results of the paired sample t-tests on patients with AT treated with the Graston technique and Alfredson procedure, as measured by the VISA-A scale, show that both groups’ symptoms significantly improved during treatment. The findings show a statistically significant rise in VISA-A scores for Group A (Alfredson procedure) from the pretreatment period to the mid-treatment phase and beyond to the posttreatment phase. These results, along with the participants’ improved capacity to participate in sports and daily activities, imply that the Alfredson regimen produced a significant improvement in AT symptoms.

The paired sample t-test also showed a significant rise in VISA-A scores for Group B (Graston technique) from the pretreatment period to the mid-treatment phase and beyond to the posttreatment phase. This may indicate that the Graston technique is effective in treating AT because it also produced a significant improvement in symptoms [27].

Overall, the paired sample t-test results offer compelling proof of the efficacy of the Graston technique and Alfredson protocol in the treatment of AT. These results bolster the usage of these treatment regimens as practical choices for people looking to reduce the symptoms related to this illness. Additional investigation, encompassing more extensive sample sizes and extended follow-up durations, could prove advantageous in validating these results and clarifying the mechanisms behind the noted ameliorations in symptoms.

One limitation of this study is the relatively small sample size, which may limit the generalizability of the findings to a broader population. A larger sample size could provide more robust evidence regarding the comparative efficacy of the Graston technique versus the Alfredson protocol in managing AT. In addition, the study’s duration of 12 weeks may not capture long-term outcomes beyond this time frame, warranting further research to assess the sustained effects of these interventions. Furthermore, the study’s focus on a single geographical location and specific demographic characteristics may limit the generalizability of the findings to other populations with different sociodemographic backgrounds or healthcare settings. Future research incorporating diverse populations and settings could enhance the external validity of the study’s findings.

## Conclusions

In this comparative study, we evaluated the efficacy of the Graston technique and Alfredson protocol in patients with AT. The results demonstrated significant improvements in VISA-A scores, indicating enhanced functional outcomes and reduced pain for both treatment groups over the 12-week intervention period. However, the Graston technique showed more significant efficacy compared to the Alfredson protocol, particularly in the mid- and post-treatment phases. These findings suggest that the Graston technique holds promise as an effective intervention for managing and alleviating symptoms associated with AT, providing valuable insights for clinicians in selecting optimal treatment modalities for this condition. In addition, patient compliance was higher with the Graston technique, which requires only two sessions per week compared to the 12-week long treatment protocol of the Alfredson method. This increased compliance, coupled with the effective soft tissue mobilization provided by the Graston technique, contributed to better overall outcomes and reduced patient dropout rates.

Future research with larger sample sizes and longer follow-up periods is warranted to corroborate these findings and establish comprehensive treatment guidelines. Comparative studies on the immediate effects of the Graston technique for other conditions, such as plantar fasciitis, should be considered. In addition, future research could compare the efficacy of the Alfredson protocol with the Silbernagel protocol for AT to further refine treatment strategies.
